# Heterozygous variants that disturb the transcriptional repressor activity of *FOXP4* cause a developmental disorder with speech/language delays and multiple congenital abnormalities

**DOI:** 10.1038/s41436-020-01016-6

**Published:** 2020-10-28

**Authors:** Lot Snijders Blok, Arianna Vino, Joery den Hoed, Hunter R. Underhill, Danielle Monteil, Hong Li, Francis Jeshira Reynoso Santos, Wendy K. Chung, Michelle D. Amaral, Rhonda E. Schnur, Teresa Santiago-Sim, Yue Si, Han G. Brunner, Tjitske Kleefstra, Simon E. Fisher

**Affiliations:** 1 Human Genetics Department, Radboud University Medical Center, Nijmegen, The Netherlands; 2 Language & Genetics Department, Max Planck Institute for Psycholinguistics, Nijmegen, The Netherlands; 3 Donders Institute for Brain, Cognition and Behaviour, Radboud University Medical Center, Nijmegen, The Netherlands; 4 Department of Pediatrics, Division of Medical Genetics, University of Utah, Salt Lake City, UT USA; 5 Department of Pediatrics, Naval Medical Center, Portsmouth, VA USA; 6 Department of Human Genetics, Emory University, Atlanta, GA USA; 7 Department of Genetics, Joe DiMaggio Children’s Hospital, Hollywood, FL USA; 8 Charles E. Schmidt College of Medicine, Florida Atlantic University, Boca Raton, FL USA; 9 Department of Pediatrics, Columbia University Irving Medical Center, New York, NY USA; 10 HudsonAlpha Institute for Biotechnology, Huntsville, AL USA; 11 GeneDx, Gaithersburg, MD USA; 12 Department of Clinical Genetics, MHeNS School of Neuroscience, and GROW-School for Oncology and Developmental Biology, Maastricht University Medical Center, Maastricht, The Netherlands; 13 Donders Institute for Brain, Cognition & Behaviour, Radboud University, Nijmegen, The Netherlands

**Keywords:** *FOXP4*, de novo variants, neurodevelopmental disorder, speech/language disorder, congenital diaphragmatic hernia

## Abstract

**Purpose:**

Heterozygous pathogenic variants in various *FOXP* genes cause specific developmental disorders. The phenotype associated with heterozygous variants in *FOXP4* has not been previously described.

**Methods:**

We assembled a cohort of eight individuals with heterozygous and mostly de novo variants in *FOXP4*: seven individuals with six different missense variants and one individual with a frameshift variant. We collected clinical data to delineate the phenotypic spectrum, and used in silico analyses and functional cell-based assays to assess pathogenicity of the variants.

**Results:**

We collected clinical data for six individuals: five individuals with a missense variant in the forkhead box DNA-binding domain of FOXP4, and one individual with a truncating variant. Overlapping features included speech and language delays, growth abnormalities, congenital diaphragmatic hernia, cervical spine abnormalities, and ptosis. Luciferase assays showed loss-of-function effects for all these variants, and aberrant subcellular localization patterns were seen in a subset. The remaining two missense variants were located outside the functional domains of FOXP4, and showed transcriptional repressor capacities and localization patterns similar to the wild-type protein.

**Conclusion:**

Collectively, our findings show that heterozygous loss-of-function variants in *FOXP4* are associated with an autosomal dominant neurodevelopmental disorder with speech/language delays, growth defects, and variable congenital abnormalities.

## INTRODUCTION

The FOXP subgroup of transcription factors consists of four different proteins: FOXP1, FOXP2, FOXP3, and FOXP4, all with important regulatory functions in developmental processes.^1–3^ For three of these FOXP proteins, heterozygous loss-of-function variants have been shown to cause Mendelian disorders, encompassing a broad spectrum of associated phenotypes. Variants in *FOXP1* cause an intellectual disability syndrome with speech delays, autism spectrum disorder, dysmorphisms, and congenital abnormalities in some affected individuals (MIM 613670);^4^ variants in *FOXP2* give rise to a disorder in which childhood apraxia of speech is a prominent feature (MIM 602081);^5^ while variants in *FOXP3* can cause X-linked immunodysregulation, polyendocrinopathy, and enteropathy (MIM 304790).^6^

In contrast to the other *FOXP* genes*, FOXP4* has not yet been convincingly linked to a Mendelian disorder. *FOXP4* is expressed in subsets of cells in a variety of tissues throughout the body, including in the developing brain, lungs, and gut.^2,7^ The encoded protein has regulatory roles in the development and maturation of the central nervous system.^8,9^ It is coexpressed with FOXP1 and/or FOXP2 in several different brain regions, such as the cortex, cerebellum, and striatum,^10^ where these transcription factors may heterodimerize, to potentially coregulate downstream targets. The phenotype associated with heterozygous germline *FOXP4* variants remains to be defined. A homozygous loss-of-function variant in *FOXP4* was previously reported in a child with developmental delays, laryngeal hypoplasia, feeding difficulties, and a ventricular septal defect, suggesting autosomal recessive inheritance.^11^ However, several different heterozygous de novo *FOXP4* variants of unknown significance have been identified in research cohorts that included individuals with specific disorders (developmental disorders, congenital diaphragmatic hernia, or high myopia)^12–14^ and in clinical diagnostic next-generation sequencing laboratories, fitting a possible autosomal dominant disease model.

We aimed to study if heterozygous de novo *FOXP4* variants can cause a specific human disorder by collecting clinical data of individuals with rare coding *FOXP4* variants, characterizing the associated phenotype, and investigating the functional impact of variants using cell-based assays. A better understanding of pathogenicity of different *FOXP4* variants and the associated disease models might directly improve clinical care by facilitating correct classification of variants found in diagnostic and research-based sequencing studies and providing families with precise recurrent risks. In addition, research on rare *FOXP4* variants and the associated phenotypes expands our knowledge of the key roles that FOXP transcription factors play in human disease.

## MATERIALS AND METHODS

### Identification and clinical characterization of individuals with *FOXP4* variants

We used GeneMatcher^15^ and denovo-db^16^ to identify individuals with de novo variants in the coding region of *FOXP4* (including canonical splice sites) and individuals with reported *FOXP4* variants of unknown significance in diagnostic next-generation sequencing studies. De-identified clinical data and variant details were collected using Castor EDC.^17^ Additional single-nucleotide variants and copy-number variants considered to be possibly pathogenic and/or to possibly contribute to the phenotype, are listed in Table S1. All variants in this paper are annotated with respect to the NM_001012426.1 transcript (FOXP4 isoform 1).

### Cell culture and transfection

HEK293T/17 cells (CRL-11268, ATCC) were cultured in DMEM (Gibco) with 10% fetal bovine serum (Gibco) and Pen/Strep (Thermo Fisher) at 37 °C with 5% CO_2_. GeneJuice (Merck Millipore) was used for transfection, following the manufacturer’s protocol.

### DNA constructs and site-directed mutagenesis

Wild-type FOXP4 (NM_138457.2; FOXP4 isoform 2) was amplified from human fetal brain complementary DNA (cDNA) using the primers listed in Table S2. Isoform 2 (NM138457.2; 667 amino acids) is a slightly shorter isoform than isoform 1 (NM_001012426.1; 680 amino acids). For consistency, all variants in this study are annotated using isoform 1. Constructs carrying *FOXP4* variants were generated using the QuikChange Lightning Site-Directed Mutagenesis Kit (Agilent). Primer sequences used for site-directed mutagenesis are provided in Table S2. FOXP4 wild-type and variant cDNAs were subcloned into pYFP and pRluc vectors (Clontech) using BamHI and XbaI restriction sites. All constructs were verified by Sanger sequencing. Plasmid sequences are available upon request.

### Luciferase assays

For the luciferase assays, we used a pGL4.23 firefly luciferase reporter vector (Promega), in which the promoter region of *SRPX2* was subcloned as previously described.^18^ HEK293T/17 cells were transfected with this firefly reporter construct (9.45 ng), a FOXP4-YFP-expression construct or empty YFP-expression vector (41.36 ng), and a pGL4.74 (hRluc/TK) Renilla reniformis luciferase construct (0.30 ng) 24 hours after seeding in 96-well plates. At 24 hours post-transfection, cells were lysed and luciferase activities were measured using the Dual-Luciferase Reporter Assay System (Promega) and an Infinite M Plex microplate reader (Tecan). Firefly luciferase activities (experimental condition) were normalized to Renilla luciferase activities (control condition).

### Fluorescence imaging of subcellular localization

HEK293T/17 cells were grown on coverslips coated with poly-D-lysine (Merck Millipore) in a 24-well plate, and transfected 24 hours after seeding, with 125 ng DNA per well. At 24 hours post-transfection, the cells were fixed with 4% paraformaldehyde (Electron Microscopy Sciences) in PBS for 15 minutes at room temperature. Hoechst 33342 (Invitrogen) was used for nuclear staining, before mounting with Fluorescence Mounting Medium (Dako).

### Bioluminescence resonance energy transfer assays

Bioluminescence resonance energy transfer (BRET) assays were performed as previously described.^19^ HEK293T/17 cells were plated in white 96-well plates with transparent bottoms (Greiner) and transfected with equimolar concentrations of YFP and RLuc plasmids. A RLuc-NLS (nuclear localization signal) plasmid was used as a negative control. At 40 hours post-transfection, medium was replaced with DMEM without phenol red and 10% fetal bovine serum (both Invitrogen), supplemented with 60 µM EnduRen Live Cell Substrate (Promega) and incubated for four hours at 37 °C. An Infinite F200PRO Microplate reader (TECAN) was used for the measurements using the Blue1 and Green1 filter. Corrected BRET ratios were calculated using the following formula: [Green1_(experimental condition)_/Blue1_(experimental condition)_] − [Green1_(control condition)_/Blue1_(control condition)_], with only the RLuc-NLS plasmid expressed in the control condition.

### Statistical analysis

For protein expression experiments, quantified microscopy data, luciferase reporter assays, and BRET assays, statistical analysis was done for each type of assay using one-way analysis of variance (ANOVA) followed by Bonferroni correction for the number of conditions tested. All analyses were performed with GraphPad Prism software.

### Ethics statement

All experiments were performed in accordance with relevant guidelines and regulations. All study proceedings involving humans were in compliance with the principles set out in the Declaration or Helsinki. Next-generation sequencing in this study was either performed in a diagnostic setting (with relevant clinical quality accreditations and consent procedures) or in a research setting (University of Alabama at Birmingham Institutional Review Board [IRB-300000328] and Columbia University Irving Medical Center Institutional Review Board [IRB-AAAB2063]). For all individuals in this study, written consent was obtained for publication of the data. For the individuals of which photos are published, specific consent for publication of photos was obtained.

## RESULTS

### Identification of *FOXP4* variants

**Fig. 1:**
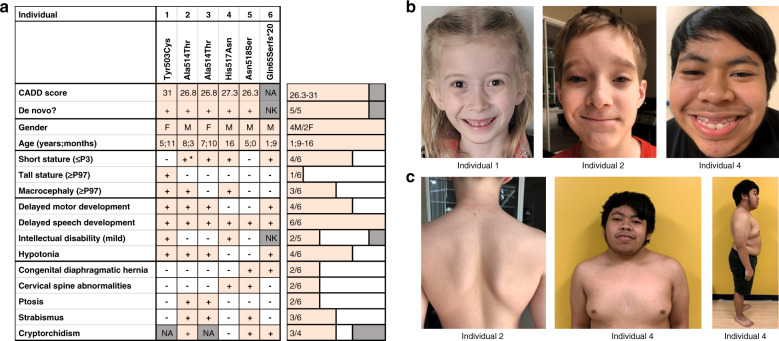
Clinical features and dysmorphisms. (**a**) Visual overview of clinical features present in six individuals with a heterozygous *FOXP4* variant, more details on phenotypes are provided in Table S1. + present, - not present, *NA* not applicable, *NK* not known. *Short stature in history, after growth hormone treatment now normal height. (**b**) Facial phenotype of three individuals with a *FOXP4* variant. Recurrently reported dysmorphisms include tented and/or flared eyebrows, ptosis, small teeth, and gingival hyperplasia. (**c**) Additional abnormalities as noted by physical examination. In individual 2, asymmetric scapulae were reported. Individual 4 presented with a very short stature (<P1) and a short and broad neck.

Using denovo-db^16^ and GeneMatcher,^15^ we aimed to collect data on all reported de novo variants in the coding region of *FOXP4* in research cohorts, as well as all reported *FOXP4* variants of unknown significance in diagnostic sequencing cohorts. Eight unrelated individuals with heterozygous *FOXP4* variants were identified, seven of whom had a de novo missense variant. One individual carried a truncating *FOXP4* variant that was not inherited from the mother; the father was unavailable for testing (Fig. 1a; Table S1). Among the seven individuals with a de novo missense variant, six different variants were found; two unrelated individuals had the same variant (p.Ala514Thr). None of the variants included in our study were present in the gnomAD and dbSNP databases.

### Phenotypes of individuals with heterozygous *FOXP4* variants

We were able to collect further details on phenotypes for six of the eight individuals with *FOXP4* variants: five individuals with a missense variant in the forkhead box DNA-binding domain (four different variants, one recurrent), and the individual with a heterozygous truncating variant (p.Gln65Serfs*20). A summary of recurrent clinical features for these six individuals can be found in Fig. 1a, with a more detailed overview in Table S1. For the two remaining individuals, both of whom had a missense variant outside the forkhead box domain, we were not able to collect additional information on phenotypes (all available data are included in Table S1).

The six individuals included in our phenotypic comparison comprised four males and two females, with an age range of 1 year 9 months to 16 years. Four individuals had a short stature (≤P3), one of these four reached a normal height after treatment with growth hormone. One individual had a tall stature. Macrocephaly (head circumference ≥ P97) was seen in three out of six individuals. Weights were generally normal for height, although one individual (individual 3) had a low weight (≤P3).

Developmental delays were observed in all six individuals. While only four of six individuals showed delayed motor development, speech/language development was delayed in all of them. All six individuals had received speech therapy and two individuals had a formal diagnosis of expressive language disorder. Despite having shown prominent speech delays in infancy, for three of six individuals (aged 5–16 years) current speech is described as normal with full and complex sentences. Two individuals had a mild intellectual disability, three individuals had no intellectual disability, and for one individual this was unknown. Infant hypotonia was seen in four of the six individuals.

Different types of congenital abnormalities were present in different individuals. Interestingly, congenital diaphragmatic hernia was present in two individuals. Vertebral abnormalities were present in two individuals: one individual had abnormalities of the craniocervical junction and malformations of several arches of C1, C2, and C3 vertebrae (details in Table S1) and in the other individual vertebra C1 was fused to the skull. An additional individual had uneven scapulae, but normal spine films (Fig. 1c). Pectus excavatum was reported in another individual. Two individuals had ptosis (requiring surgery in one individual), and strabismus was reported in three individuals. Cryptorchidism was present in three of four males. In addition to congenital abnormalities, overlapping facial features were reported in several individuals, which included tented and/or flared eyebrows, small teeth, and gingival hyperplasia (Fig. 1b).

### In silico variant analysis

**Fig. 2:**
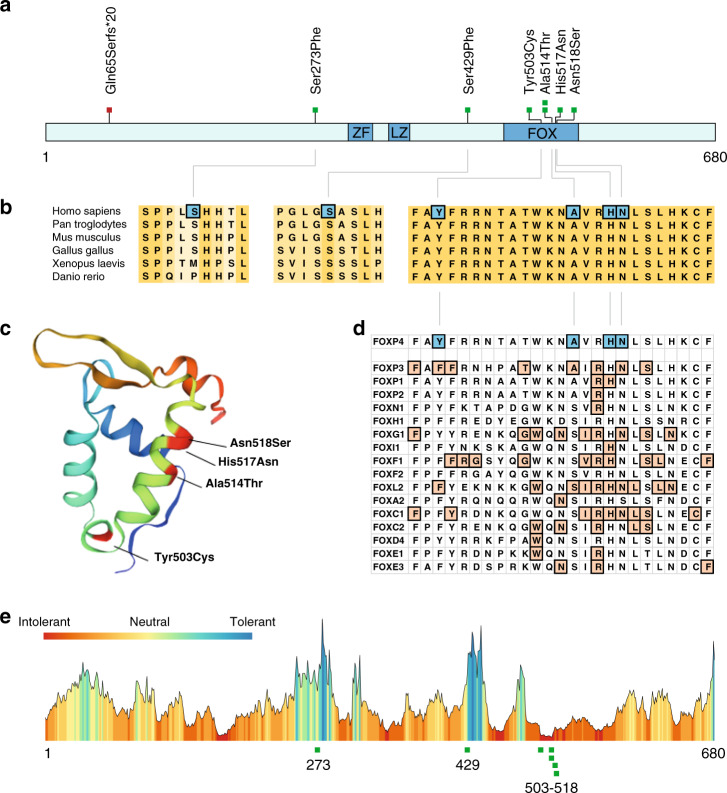
In silico analyses of heterozygous *FOXP4* variants. (**a**) Linear representation of the FOXP4 protein (Q8IVH2–1) with the identified variants and functional domains annotated: *FOX* forkhead box domain, *LZ* leucine zipper, *ZF* zinc finger. (**b**) Conservation of FOXP4 across different species, with the amino acids affected by missense variants indicated. Species include *Homo sapiens* (UniProt sequence Q8IVH2), *Pan troglodytes* (A0A2J8NZN5), *Mus musculus* (Q9DBY0), *Gallus gallus* (A0A3Q2U1E5), *Xenopus laevis* (Q4VYR7), and *Danio rerio* (B3DJK9). Regions shown span amino acids 269–277, 425–433, and 501–525 of FOXP4 isoform 1 (Q8IVH2). (**c**) Visualization of missense variants in the FOX domain in a three-dimensional structure. A homology model for the FOX domain of FOXP4 (amino acids 456–542) was built based on template structure 2kiu.1.A (FOXP1 monomer), using the SWISS-MODEL Homology Modeling online tool.^20^ (**d**) Alignment of missense variants in a subset of the FOX domain with pathogenic missense variants in other FOX proteins. An alignment was made of the Pfam Forkhead domain (PF00250) using Clustal Omega Multiple Sequence Alignment^44^ of all FOX proteins with missense variants present in HGMD database 2019.3. Only missense variants labeled as pathogenic were included for this analysis. (**e**) Tolerance landscape of FOXP4 protein visualized via the MetaDome web server.^23^ A tolerance landscape is computed based on single-nucleotide variants in the gnomAD database, and shows per amino acid position the missense over synonymous ratio in a sliding window of 21 residues. Green and blue peaks represent regions tolerant to missense variation; red valleys show intolerant regions. The missense variants in the FOX domain are located in extremely intolerant regions of FOXP4, while the two remaining missense variants are located in extremely tolerant regions.

We used an array of computational tools to predict the functional effects of all missense variants that were found, including the two missense variants for which no additional information on phenotypes was available. Four of the six different missense variants clustered in the DNA-binding forkhead box domain of the encoded FOXP4 protein, while the remaining two were located outside known functional domains (Fig. 2a). The cross-species conservation of the amino acid sequences in the affected regions is shown in Fig. 2b. The mutated amino acid sites are invariant across all the species that we analyzed, with the sole exception of the Serine 273 residue, which is less conserved. For all missense variants, CADD, PolyPhen, and SIFT scores were derived, all of which predicted pathogenicity for the four forkhead box domain variants (Fig. 1a and Table S1).

As no three-dimensional protein structure is available for FOXP4, we used the SWISS-MODEL Homology Modeling online tool^20^ to create a homology model of the forkhead box domain structure of FOXP4 (amino acids 456–542) based on a FOXP1 template model. We then visualized the three-dimensional location of the four different missense variants mapping to this functional domain (Fig. 2c). Three of the four missense variants (p.Ala514Thr, p.His517Asn, p.Asn518Ser) are located in the third helix of the DNA-binding domain (the recognition helix), and the fourth variant (p.Tyr503Cys) maps to the hinge loop region.

FOXP4 belongs to the large family of FOX transcription factor proteins, defined by the presence of the distinctive highly conserved forkhead box domain. For at least 16 FOX proteins, missense variants in this characteristic DNA-binding domain have already been linked to Mendelian disorders in humans.^21,22^ We therefore assessed whether the potentially pathogenic missense variants that we identified in the forkhead box domain of FOXP4 were comparable with the known pathogenic missense variants in these other FOX transcription factors (Fig. 2d). Indeed missense variants in the FOXP4 DNA-binding domain matched well to the known pathogenic missense variants in other FOX transcription factors (Fig. 2d).

We went on to use the MetaDome web tool^23^ to visualize all six different *FOXP4* missense variants in the tolerance landscape of the gene (Fig. 2e), which shows regional tolerance for genetic variation based on a missense over synonymous variant count ratio using data from the gnomAD database.^24^ This showed us that the four missense variants that cluster in the FOX domain are located in a region of high intolerance (low missense over synonymous variant count ratio), while the two that map elsewhere are located in more tolerant regions of the protein (see Table S1). It is interesting to note that the gnomAD *Z*-score for missense variants in *FOXP4* as a whole is not particularly high (1.95),^24^ indicating that the complete coding region of the *FOXP4* gene is not extremely intolerant for missense variation overall. This finding is in line with the results from the MetaDome analysis, which show that only a few small regions of *FOXP4* show high intolerance for missense variants, including the part of the forkhead box domain in which our four different missense variants are located.

### Effects of variants on localization and transcriptional repression activity

**Fig. 3:**
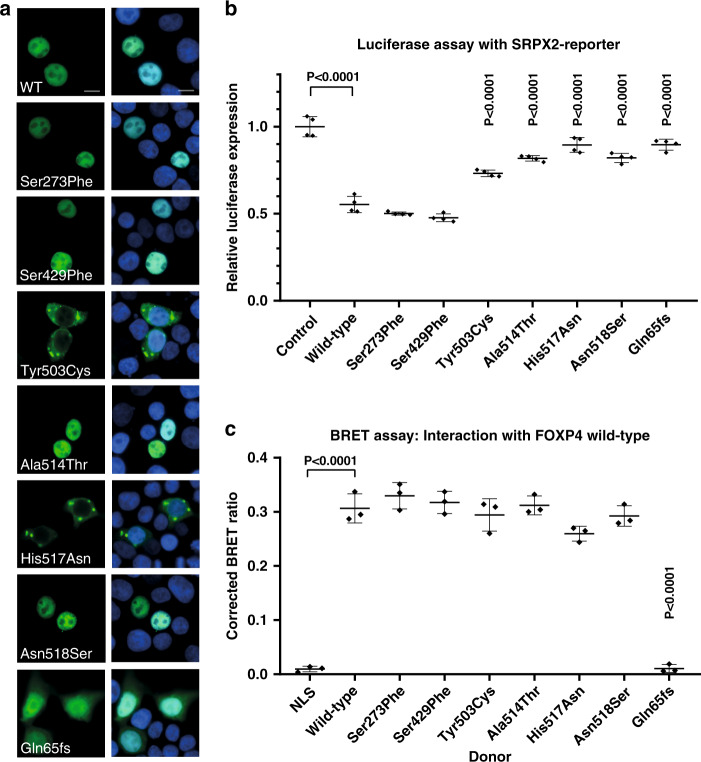
Functional assays to assess pathogenicity. (**a**) Direct fluorescence imaging of HEK293T/17 cells expressing YFP-FOXP4 fusion proteins (green) with the different FOXP4 variants found in our cohort. Nuclei are stained with Hoechst 33342. Scale bar = 10 µm. (**b**) Results of luciferase assays with FOXP4-YFP constructs and the SRPX2-reporter construct. Values are expressed relative to the control construct and represent the mean ± SD of four independent experiments, each performed in triplicate. *P* values were calculated using one-way analysis of variance (ANOVA) with Bonferroni correction. (**c**) Results of bioluminescence resonance energy transfer (BRET) assays to measure dimerization capacity of mutant FOXP4 constructs (donor) with wild-type (WT) FOXP4 (acceptor). Values represent the corrected mean BRET ratio ± SD of three independent experiments performed in triplicate. *P* values were calculated using one-way ANOVA with Bonferroni correction.In panel A, B and C, ‘Gln65fs’ is used as a short description for the variant ‘p.Gln65Serfs*20’.

Functional assays in HEK293T/17 cells were performed for all the seven different FOXP4 variants that were identified (six missense variants and one variant causing an early frameshift). We used overexpression constructs of FOXP4 (isoform 2) with an N-terminal YFP-tag to assess the subcellular localization of the respective mutant FOXP4 proteins. While all experiments were performed with isoform 2 FOXP4 proteins (consisting of 667 amino acids), all variants in this study are annotated in isoform 1 for consistency of the interpretation. Immunoblotting indicated that the wild-type and mutant proteins were expressed at the expected size and at comparable levels (Fig. S1). In assessments of subcellular localization using fluorescence imaging, wild-type FOXP4 showed nuclear localization, as did mutant proteins with the two missense variants mapping outside the known functional domains (p.Ser273Phe and p.Ser429Phe; Fig. 3a). Three of the four different missense variants in the forkhead box domain led to aberrant localization of the mutant protein: p.Tyr503Cys and p.His517Asn showed cytoplasmic expression with aggregates, and for the p.Asn518Ser variant a nuclear granular pattern was seen. Overexpression of the truncated protein yielded by the frameshift variant (p.Gln65Serfs*20) led to diffuse mislocalization in the cytoplasm, although the protein was still present in the nucleus.

We used luciferase assays to assess the capacity of FOXP4 to repress an *SRPX2*-derived promoter element. Wild-type FOXP4 showed significant repression of reporter gene expression compared with a control construct (Fig. 3b). The four FOXP4 proteins with amino acid substitutions in the forkhead box domain (p.Tyr503Cys, p.Ala514Thr, p.His517Asn, and p.Asn518Ser) all showed a loss of this transcriptional repressor activity, significantly different from wild-type FOXP4. Loss of function was also seen for the truncated FOXP4 protein (p.Gln65Serfs*20), consistent with the lack of a DNA-binding domain. For proteins with the two remaining missense variants (p.Ser273Phe and p.Ser429Phe), both located outside known functional domains of FOXP4, repression capacities were no different from the wild-type protein. In summary, the localization and luciferase assays pointed to pathogenicity for the four missense variants in the forkhead box domain, with a loss-of-function mechanism, in contrast to the two missense variants located elsewhere in the protein, which did not differ from wild-type in these experiments.

As FOXP4 is known to be able to dimerize with itself and/or other FOXP proteins, mediated by a conserved leucine zipper motif,^25^ we also assessed the effects of variants on dimerization capacities using BRET assays. In these assays, wild-type and variant versions of FOXP4 with an N-terminal Renilla luciferase tag (donor) were coexpressed with wild-type FOXP4 with an N-terminal YFP-tag (acceptor). The corrected BRET ratios of all FOXP4 proteins with missense variants were no different from those of wild-type FOXP4, indicating intact dimerization capacities for all these proteins (Fig. 3c). The truncated version of FOXP4 showed a complete loss of dimerization capacity, similar to the negative rLuc-NLS control construct (Fig. 3c).

## DISCUSSION

To characterize the clinical and molecular consequences of heterozygous *FOXP4* variants identified in several next-generation sequencing cohorts, we collected data on individuals with rare and possibly pathogenic variants in this gene. We identified seven individuals with a de novo missense variant (six different variants, since one was found independently in two unrelated cases). Using luciferase assays, we showed that four of the six different missense variants had loss-of-function effects on transcription repressor activity of the encoded FOXP4 protein. Notably, these four disruptive missense variants were all located in the forkhead box DNA-binding domain, a key functional motif of the protein. There was also one individual with a frameshift variant of unknown parental origin. The transcript with the frameshift variant will most likely undergo nonsense-mediated decay (NMD), leading to *FOXP4* haploinsufficiency in this individual, and our cell-based experiments indicate that any truncated protein resulting from NMD escape would lack repressor activity. Based on our findings we conclude that heterozygous *FOXP4* variants can cause a neurodevelopmental disorder, with prominent speech/language problems, short stature, macrocephaly, overlapping dysmorphisms, congenital diaphragmatic hernia, and cervical vertebral abnormalities.

Four of the six different missense variants were clustered in the DNA-binding domain of the encoded protein, at positions that are highly conserved across species, and also across different members of the FOX transcription factor family (Fig. 2b, d). Three of these four missense variants (p.Ala514Thr, p.His517Asn, and p.Asn518Ser) map within the third helix of the DNA-binding domain (Fig. 2c), also known as the recognition helix since it mediates sequence specific interaction with nucleotides in the major groove of the DNA of downstream targets.^26^ Many different missense variants in FOX family proteins at similar positions in the DNA-binding domain have already been shown to be pathogenic (Fig. 2d). Using direct immunofluorescence, we showed that three of the four missense variants located in the DNA-binding domain of FOXP4 led to an aberrant subcellular localization of the protein. Although the precise mechanism by which these variants affect the localization pattern is not known, these results match well with previous observations of aberrant localization patterns associated with missense variants in the FOX domain of *FOXP1*^27^ and *FOXP2*,^28^ and in more distantly related forkhead genes such as *FOXC1*^29^ and *FOXC2*.^30^ We used luciferase assays with an SRPX2-derived promoter sequence as a reporter to demonstrate that each variant yielded a loss of transcriptional repression activity for the respective FOXP4 protein. The fourth DNA-binding domain variant, p.Tyr503Cys, is located in the hinge loop region of this motif. Previous studies reported that a variant of the conserved tyrosine residue at the equivalent position in FOXP2 (p.Tyr540Phe in FOXP2 isoform 1; NP_055306.1) disrupted DNA binding, and also had effects on dimerization capability.^31^ In our assays, the p.Tyr503Cys variant of FOXP4 significantly disrupted transcription factor capacities to a similar degree to the other DNA-binding domain variants, consistent with loss of function, but no effect on dimerization with FOXP4 wild-type protein was observed. All in all, the observations in functional studies for the different forkhead box DNA-binding domain missense variants all point to a loss-of-function effect, which is in line with existing literature about other *FOX*-associated disorders.

Two missense variants (p.Ser273Phe and p.Ser429Phe) did not show any difference compared with wild-type FOXP4 in functional assays. For these variants, the functions of the regions and amino acids involved is not known. The p.Ser273Phe variant was found in a large exome sequencing study in children with developmental disorders^14^ but no additional information on phenotype could be collected for this individual. The p.Ser429Phe variant was found in a small trio exome sequencing cohort, in a young child with high myopia.^13^ This individual also carried a hemizygous missense variant in *CACNA1F* (NP_005174.2: p.[Arg1060Trp]), which has already been described as a pathogenic variant causing X-linked congenital stationary night blindness (MIM 300071), possibly explaining the phenotype in this individual. Taking all data into account, these two missense variants might very well be benign variants, although pathogenicity cannot be completely excluded based on our assays.

The remaining variant in our study was a frameshift variant, for which the transcript will most likely undergo NMD, resulting in haploinsufficiency for FOXP4. If the transcript with the variant would still escape NMD, a truncated and dysfunctional version of FOXP4 would be expressed that has an aberrant subcellular localization pattern, does not show transcriptional repressor capacities in *SRPX2*-reporter luciferase assays, and is unable to dimerize. *FOXP4* is known to be extremely intolerant of loss-of-function variation, with a probability of loss-of-function intolerance (pLI) score of 0.98 based on sequencing data from 141,456 individuals, providing independent evidence that *FOXP4* haploinsufficiency is pathogenic.^24^ The Decipher database contains seven microdeletions encompassing *FOXP4*, but as these are all large deletions (2.41 to 4.57 Mb in size) it is hard to draw conclusions about the contribution of *FOXP4* haploinsufficiency to the corresponding phenotypes. But interestingly, in the literature one individual has been reported with developmental delays, laryngeal hypoplasia and a ventricular septal defect, and a homozygous truncating *FOXP4* variant: c.815del; p.(Leu272Profs*95).^11^ Both parents were shown to be heterozygous for this variant, suggesting autosomal recessive inheritance, but no further clinical details were reported on the parents or other family relatives. As the individual with the heterozygous frameshift variant in our cohort had a phenotype entirely in line with the individuals with the likely pathogenic forkhead box domain missense variants: a congenital diaphragmatic hernia, short stature, developmental delays, hypotonia, and cryptorchidism, we assume that the *FOXP4* variant is causative. Although for our missense variants we cannot exclude a possible dominant-negative mechanism in addition to the loss-of-function effects, in which FOXP4 proteins with these variants would interfere with wild-type FOXP protein functions via their intact dimerization capacities, we propose that truncating variants in FOXP4 can be pathogenic in a heterozygous state.

*FOXP4* was first characterized by Lu et al.^2^ and Teufel et al.^32^ and shown to be expressed in a range of tissues, including heart, brain, lung, liver, kidney, and testis. Importantly, *FOXP4* is not only expressed in adult tissue, but also during different stages of development of, e.g., the heart, lungs, gut, and skeleton, where it has been shown to play important functional roles.^2,32–35^ This widespread expression pattern, in combination with the large number of transcriptional targets and protein–protein interactions known for FOXP transcription factors,^10,36–38^ could potentially yield a large variety of downstream consequences when FOXP4 functions are compromised. It is thus not surprising that we found a broad range of associated phenotypes in individuals with likely pathogenic *FOXP4* variants, including growth deficits, developmental delays and a spectrum of associated congenital abnormalities. Although caution is warranted given the limited cohort size of our study, variants in *FOXP4* seem to be associated with certain phenotypic features (e.g., vertebral abnormalities and congenital diaphragmatic hernia) that appear distinct from those observed in individuals carrying variants in *FOXP1* or *FOXP2*. Congenital anomalies are not a common finding in individuals with pathogenic *FOXP2* variants,^39^ and in *FOXP1*-associated disorder different abnormalities are recurrently reported, such as congenital heart defects or kidney abnormalities^40,41^ (Table S3). Variants in *FOXP3* are not associated with a neurodevelopmental disorder phenotype, and were thus not included in this phenotypic comparison. Future studies will establish how the distinctive FOXP expression patterns, together with differences in profiles of cofactors and downstream targets in the relevant tissues, contribute to the different phenotypes associated with haploinsufficency of each transcription factor.

Of note, in *FOXP1-* and *FOXP2*-related disorders, expressive speech problems are a prominent feature,^40^ and the contributions of these regulatory factors to the development and function of relevant neural circuits are extensively studied.^10,42^ A recent study linking FoxP1/2/4 functions to vocal learning in songbirds suggested that FOXP4 should also be considered as a candidate for involvement in vocal disorders.^43^ Indeed, all individuals with likely pathogenic FOXP4 variants in our study had delayed speech/language development, with expressive problems prominently present. As FOXP1, FOXP2, and FOXP4 show partially overlapping coexpression in various different regions of the developing brain,^10^ further research is needed to delineate if loss-of-function of FOXP4 directly impairs speech/language development, or whether secondary disruption of FOXP1 and/or FOXP2 function via heterodimerization with dysfunctional FOXP4 could play a role as well.

In conclusion, through clinical characterization and functional assays, we implicate heterozygous *FOXP4* variants in a neurodevelopmental disorder with mild developmental delays, most prominently in the speech/language domain. The disorder shows variable expressivity: a broad spectrum of associated features is present in a subset of individuals and includes short stature, macrocephaly, congenital diaphragmatic hernia, vertebral abnormalities, ptosis, and cryptorchidism. As several congenital abnormalities are recurrently observed in our patients with likely pathogenic variants, and developmental delays can be mild, the possibility of *FOXP4* involvement should not only be considered in individuals with neurodevelopmental disorders but also in cohorts of individuals with multiple congenital abnormalities, in particular, congenital diaphragmatic hernia and/or vertebral abnormalities.

## Supplementary information

Supplementary Table S1

Supplementary Information
