# Supplementary material for: Heterozygous variants that disturb the transcriptional repressor activity of FOXP4 cause a developmental disorder with speech/language delays and multiple congenital abnormalities
Source: Genet Med. 2020 Oct 28;23(3):534–42. doi: 10.1038/s41436-020-01016-6 (PMC7935712; doi:10.1038/s41436-020-01016-6)
Supplement: Supplementary file 2 — Supplementary Information [file 41436_2020_1016_MOESM2_ESM.pdf]

Supplementary Information

Supplementary Materials and Methods

Immunoblotting

Whole-cell lysates were collected 24 hours post-transfection, as previously described[1]. The membrane was probed with 1:8000 mouse anti-EGFP (Clontech) in 1% milk in PBS-T overnight, followed by incubation with 1:3000 HRP-conjugated goat anti-mouse (Bio-Rad) in 1% milk at room temperature for 1h. After visualization using the Novex ECL Chemiluminescent Substrate Reagent Kit (Invitrogen) and the ChemiDoc XRS+ System (Bio-Rad), the blot was stripped for 20 minutes in Re-blot Plus Strong stripping solution (Millipore) and blocked in 5% milk in PBS-T for one hour. This was followed by incubation with 1:10,000 mouse anti-beta-actin (Sigma) for 1.5 hour, and incubation with 1:3000 HRP-conjugated goat anti-mouse (Bio-Rad) for one hour.

Table S2: Primer sequences

Primer	Sequence (5'-3')
FOXP4 cloning with BamHI restriction site (forward)	aggatcctggtggaatctgcctcggagac
FOXP4 cloning with XbaI restriction site (reverse)	ctctagattaggacagttcttctcccggca
Site-directed mutagenesis Y503C (forward)	caccaggatgttcgcctgtttccgcagaaacactg
Site-directed mutagenesis Y503C (reverse)	cagtgtttctgcggaaacaggcgaacatcctggtg
Site-directed mutagenesis N518S (forward)	acgccgtgcgccacagcctcagcc
Site-directed mutagenesis N518S (reverse)	ggctgaggctgtggcgcacggcgt
Site-directed mutagenesis S429F (forward)	ccctggcctgggctttgcctccctg
Site-directed mutagenesis S429F (reverse)	cagggaggcaaagcccaggccaggg
Site-directed mutagenesis Q65Sfs*20 (forward)	gagcctgttgctgctgaagtgcagcagctc
Site-directed mutagenesis Q65Sfs*20 (reverse)	gagctgctgcacttcagcagcaacaggctc
Site-directed mutagenesis S273F (forward)	gtctcacccccccctcttccaccataccctgc
Site-directed mutagenesis S273F (reverse)	gcagggtatggtggaagagggggggtgagac
Site-directed mutagenesis A514T (forward)	gccacctggaagaacaccgtgcgccac
Site-directed mutagenesis A514T (reverse)	gtggcgcacggtgttcttccaggtggc
Site-directed mutagenesis H517N (forward)	gaacgccgtgcgcaacaacctcagcct
Site-directed mutagenesis H517N (reverse)	aggctgaggttgttgcgcacggcgttc

Table S3: Comparison of phenotypes associated with variants in *FOXP1, FOXP2 and FOXP4*

	FOXP4	FOXP1	FOXP2
**Short stature (≤P3)**	+	+	-
**Tall stature (≥P97)**	+	-	+
**Macrocephaly (≥P97)**	+	+	+
**Delayed motor development**	+	+	+
**Delayed speech development**	+	+	+
**Intellectual disability**	+	+	-
**Hypotonia**	+	+	-
**Congenital diaphragmatic hernia**	+	-	-
**Cervical spine abnormalities**	+	-	-
**Ptosis**	+	+	-
**Strabismus**	+	+	+
**Cryptorchidism**	+	+	-
**Kidney abnormalities**	-	+	-
**Genital abnormalities**	-	+	-
**Congenital heart defect**	-	+	-
**Joint contractures/arthrogryposis**	-	+	-

The phenotypic features of individuals with FOXP4 variants in our study, in comparison with phenotypes reported in individuals with pathogenic *FOXP1* or *FOXP2* variants. For the analysis of common *FOXP1*- and *FOXP2*-associated features, the following cohort studies were used: Bekheirnia et al.2017[2], Le Fevre et al. 2013[3], Reuter et al. 2017[4], Siper et al. 2017[5], Sollis et al. 2016[6] and Sollis et al. 2017[7]. For *FOXP1* and *FOXP2*, a '+' was scored if this feature was reported in two unrelated individuals in the studies mentioned. As a result, some phenotypes annotated with a '+' are only present in a small subset of individuals with *FOXP1*- or *FOXP2*-associated disorder.

Figure S1: Immunoblot analysis of overexpression constructs

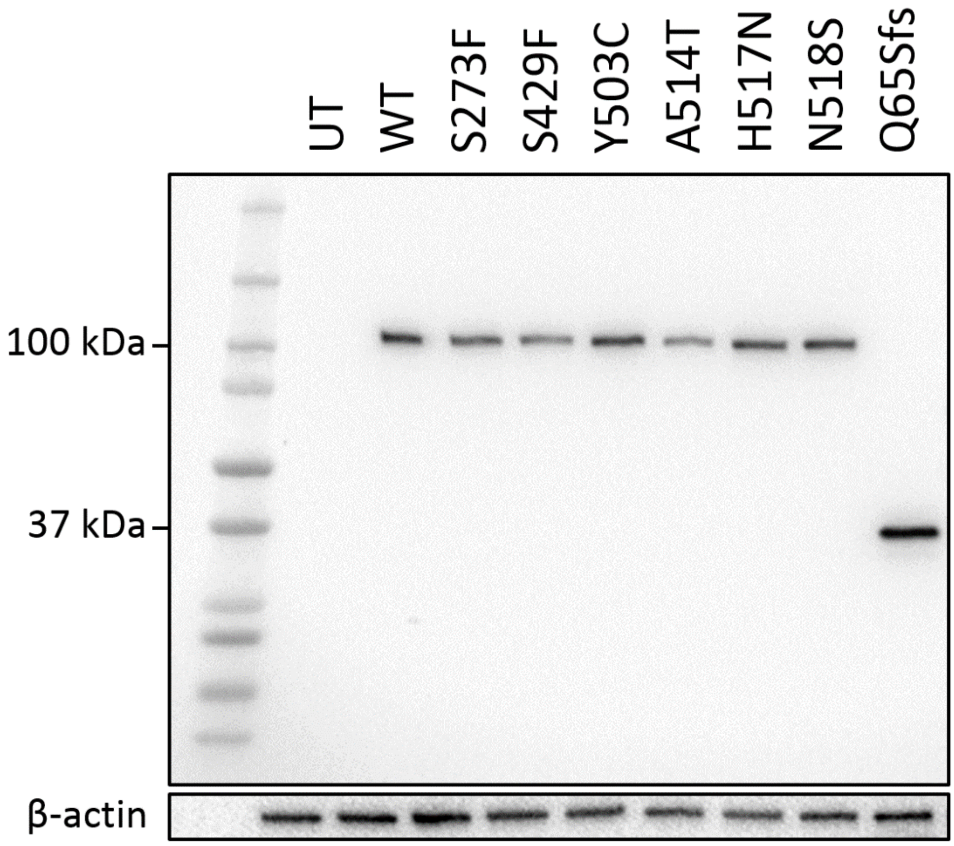

Western Blot of HEK293T/17 cells in which FOXP4-YFP constructs used for functional assays were overexpressed. UT = untransfected, WT= wild-type. Expected molecular weight of wild-type FOXP4-YFP and FOXP4-YFP with missense variants: ~102 kDa. Expected molecular weight of Q65Sfs*20 variant: ~38kDa. All different expressed YFP-fusion proteins are present at the expected molecular weights. The immunoblot was stripped and re-probed with beta-actin as a loading control.

Supplementary References

1. Snijders Blok L, Kleefstra T, Venselaar H, et al. De Novo Variants Disturbing the Transactivation Capacity of POU3F3 Cause a Characteristic Neurodevelopmental Disorder. *Am J Hum Genet.* 2019;105(2):403-412.
2. Bekheirnia MR, Bekheirnia N, Bainbridge MN, et al. Whole-exome sequencing in the molecular diagnosis of individuals with congenital anomalies of the kidney and urinary tract and identification of a new causative gene. *Genet Med.* 2017;19(4):412-420.
3. Le Fevre AK, Taylor S, Malek NH, et al. FOXP1 mutations cause intellectual disability and a recognizable phenotype. *Am J Med Genet A.* 2013;161A(12):3166-3175.
4. Reuter MS, Riess A, Moog U, et al. FOXP2 variants in 14 individuals with developmental speech and language disorders broaden the mutational and clinical spectrum. *J Med Genet.* 2017;54(1):64-72.
5. Siper PM, De Rubeis S, Trelles MDP, et al. Prospective investigation of FOXP1 syndrome. *Mol Autism.* 2017;8:57.
6. Sollis E, Graham SA, Vino A, et al. Identification and functional characterization of de novo FOXP1 variants provides novel insights into the etiology of neurodevelopmental disorder. *Hum Mol Genet.* 2016;25(3):546-557.
7. Sollis E, Deriziotis P, Saitsu H, et al. Equivalent missense variant in the FOXP2 and FOXP1 transcription factors causes distinct neurodevelopmental disorders. *Hum Mutat.* 2017;38(11):1542-1554.
